# Camera trapping survey of vertebrates in Lipis Geopark, Pahang, Malaysia with notes on cave utilisation by mainland clouded leopard (*Neofelis nebulosa*)

**DOI:** 10.3897/BDJ.14.e166468

**Published:** 2026-06-17

**Authors:** Siti Nurfarhana Zafirah Azidi, Nur Zakirah Halmi, Jacqueline Clara Anak Chuat, Shameera Nasreen Ahamed Noordeen, Muhammad Hafiz Mohd Yazid, Muhammad Aminuddin Baqi Hasrizal Fuad, Mohd Nur Arifuddin, Izereen Mukri, Fong Pooi Har, Anthony J. Giordano, Jayaraj Vijaya Kumaran

**Affiliations:** 1 Faculty of Earth Science, Universiti Malaysia Kelantan, Jeli, Kelantan, Malaysia Faculty of Earth Science, Universiti Malaysia Kelantan Jeli, Kelantan Malaysia; 2 Sime Darby Property, Kuala Lumpur, Malaysia Sime Darby Property Kuala Lumpur Malaysia; 3 Lik Tin Environment Consultancy, Tanah Merah, Kelantan, Malaysia Lik Tin Environment Consultancy Tanah Merah, Kelantan Malaysia; 4 Society for the Preservation of Endangered Carnivores and their International Ecological Study (S.P.E.C.I.E.S.), Ventura, California, United States of America Society for the Preservation of Endangered Carnivores and their International Ecological Study (S.P.E.C.I.E.S.) Ventura, California United States of America; 5 Center for Human-Carnivore Coexistence, Colorado State University, Fort Collins, United States of America Center for Human-Carnivore Coexistence, Colorado State University Fort Collins United States of America

**Keywords:** camera trapping, vertebrate diversity, limestone karst, Lipis Geopark, cave ecology, buffer zones, *Neofelis nebulosa*

## Abstract

Lipis Geopark, a recently designated National Geopark in Pahang, is geologically rich in limestone karst formations centred in Merapoh. Its north-eastern region comprises a portion of the Taman Negara Pahang, with an entry point at Sungai Relau, Merapoh. However, research conducted in the area is highly concentrated in the forested regions inside Taman Negara Pahang, with minimal studies assessing vertebrate diversity outside the protected National Park. Thus, this study compiled data on the diversity of vertebrates in Lipis Geopark, comparing two distinct habitats: the caves of Merapoh and the Taman Negara Pahang buffer zones. A total of 20 camera traps were installed at: (1) Site A, Merapoh cave complex and (2) Site B, forested buffer zones of Taman Negara Pahang. For about 1,100 camera trap nights of 55 days, up to 30 species comprising reptiles, aves and mammals were photo-captured from 288 independent occasions. Altogether, 7,755 photo images were captured, and only 52.174% were identified as wildlife images. The remaining images were excluded from the data analysis. It was observed that the Eurasian wild pig (*Sus scrofa*) is the most photographed species with the highest PCRI (Photographic Capture Rate Index) value of 14.000 and Site A exhibited greater species evenness and diversity compared to Site B, despite recording fewer total individuals. Additionally, a notable documentation of a mainland clouded leopard (*Neofelis nebulosa*), a Vulnerable species, utilising a cave in Merapoh has been recorded – representing a rare and possibly novel behaviour in Peninsular Malaysia. Thus, these findings highlight the ecological importance of karst caves and surrounding forests in Lipis Geopark, underscoring the urgent need for expanded biodiversity assessments and integrated conservation strategies within and beyond protected area boundaries.

## Introduction

Lipis Geopark is part of the Central Gold Belt (CGB), strategically located to the east of the contiguous and most extensive Titiwangsa mountain range and is connected to the greater Taman Negara Pahang through its Buffer Zone (BZ) (Pour and Mazlan 2015; Chan et al. 2019). The BZ concept has been proposed as an additional layer of protection, safeguarding the protected areas where biodiversity conservation is the primary objective. It serves as a dual-purpose buffering: insulating the Protected Area (PA) or core area from any external influences that potentially damage its conservation purposes; and permitting land-use activities restricted to those BZs that are economically valuable to the indigenous community in the area (Bennett and Mulongoy 2006; Regional Planning Division, 2009; PLANMalaysia 2022). Furthermore, the Lipis Geopark encompasses a section of the Central Forest Spine Master Plan, specifically the Central Forest Spine–Primary Linkage 1 (C-PL1) (PLANMalaysia 2022), an ecological corridor designed to restore connectivity between fragmented forest patches. Additionally, the Geopark features limestone karst terrain characterised by fragmented, island-like formations of caves, sinkholes and limestone towers. In Peninsular Malaysia, limestone karsts cover less than 1% of the land area, yet they foster exceptional biodiversity and endemism. Nonetheless, it is estimated that only 2% of the known karst formations and limestone forests in Peninsular Malaysia have been explored, despite their biodiversity potential (Grismer et al. 2013; Quah et al. 2021).

Situated within the Lipis Geopark, the small town of Merapoh is home to an extensive cave system estimated to be approximately 130 million years old. However, to date, fewer than half of these caves have been explored. Additionally, research on vertebrates in Malaysian karst systems has largely focused on cave-obligate species. For instance, a recent study in Merapoh has recorded 32 species of bats across several limestone caves (Hasrizal Fuad et al. 2024). There is a notable lack of studies assessing the usage of these karst ecosystems by medium- to large-bodied terrestrial vertebrates. This knowledge gap hampers conservation planning in karst regions, many of which face pressures from quarrying and land-use change. To address these research gaps at Lipis Geopark, this study aimed to document the diversity of mid- to large-sized vertebrates within the Merapoh karst complex and buffer zones at Taman Negara Pahang. By deploying systematic camera traps across limestone caves in Lipis Geopark, we provide new insights into species presence, habitat use and conservation implications for karst-associated wildlife in Peninsular Malaysia.

## Study area description

**Figure 1.**
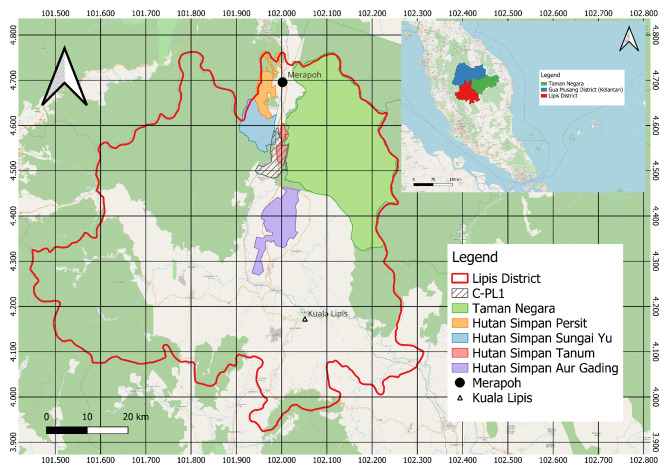
Lipis Geopark covers the whole Lipis District in Pahang, Peninsular Malaysia.

Lipis Geopark is located in the northwest of Pahang, the largest state in Peninsular Malaysia (Fig. 1). The Geopark spans the entire Lipis District, encompassing numerous permanent forest reserves and forming part of Taman Negara Pahang on its north-eastern boundary. Locally known as a caving paradise, Merapoh is situated north of the Geopark and is surrounded by numerous limestone hills of the Gua Musang Formation (Muhammad Aminuddin Baqi et al. 2020).

**Figure 2.**
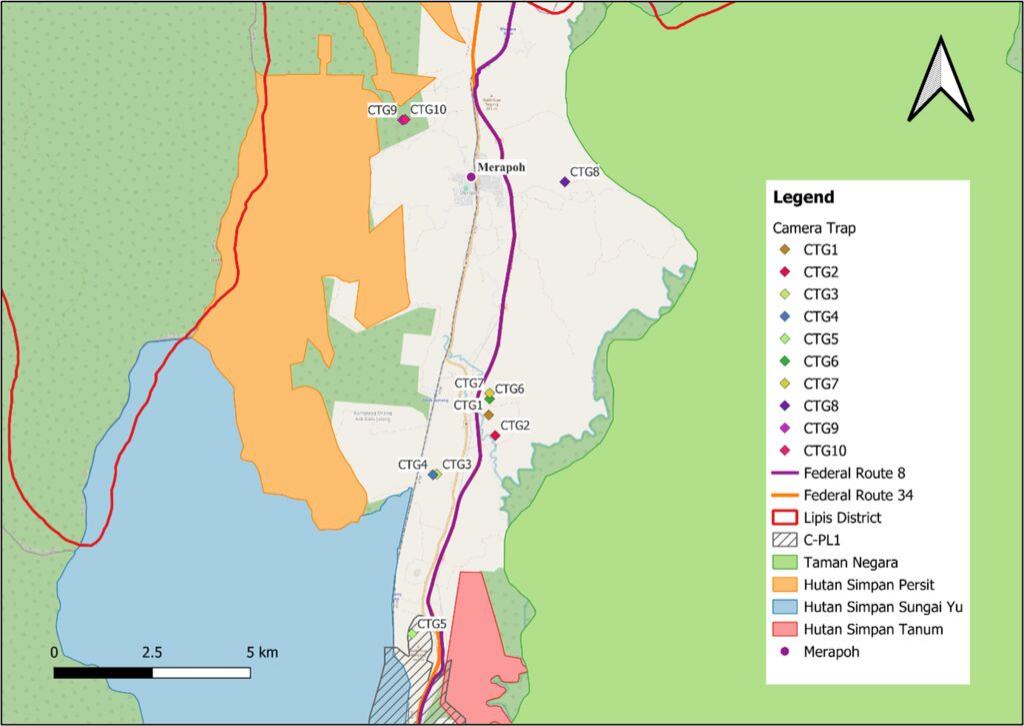
Ten camera traps were installed at the cave entrance and exit within Merapoh.

**Figure 3.**
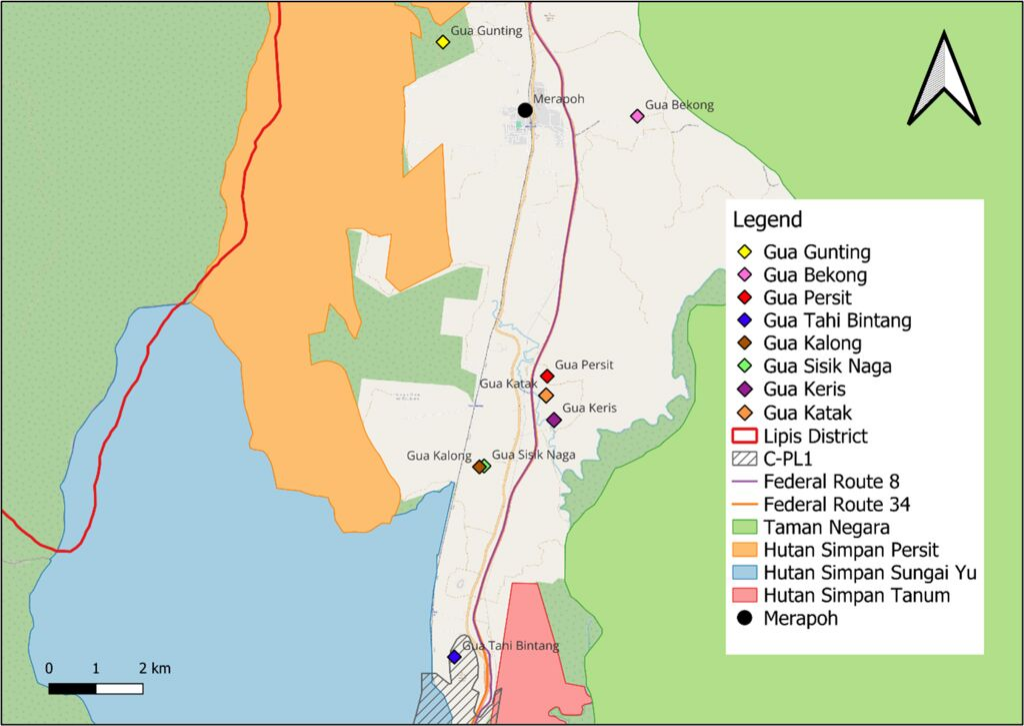
Eight caves in Merapoh sampled for this study.

Ten camera traps were strategically deployed across Site A (Fig. 2), which comprises eight caves within the Merapoh cave complex (Fig. 3). These caves are situated along Federal Route 34, the Lingkaran Tengah Utama Expressway (formerly the Central Spine Road) and exhibit varying degrees of anthropogenic influence and surrounding land use. Gua Persit and Gua Katak are located within mixed plantations and fruit orchards. Areas surrounding G. Bekong and G. Tahi Bintang are progressively cleared, presumably for agricultural expansion. G. Kalong, G. Sisik Naga and G. Keris occur parallel to plantations, bordered by forest patches dominated by secondary vegetation. In contrast, G. Gunting is located within a forested landscape, maintaining ecological connectivity with Hutan Simpan Persit.

**Figure 4.**
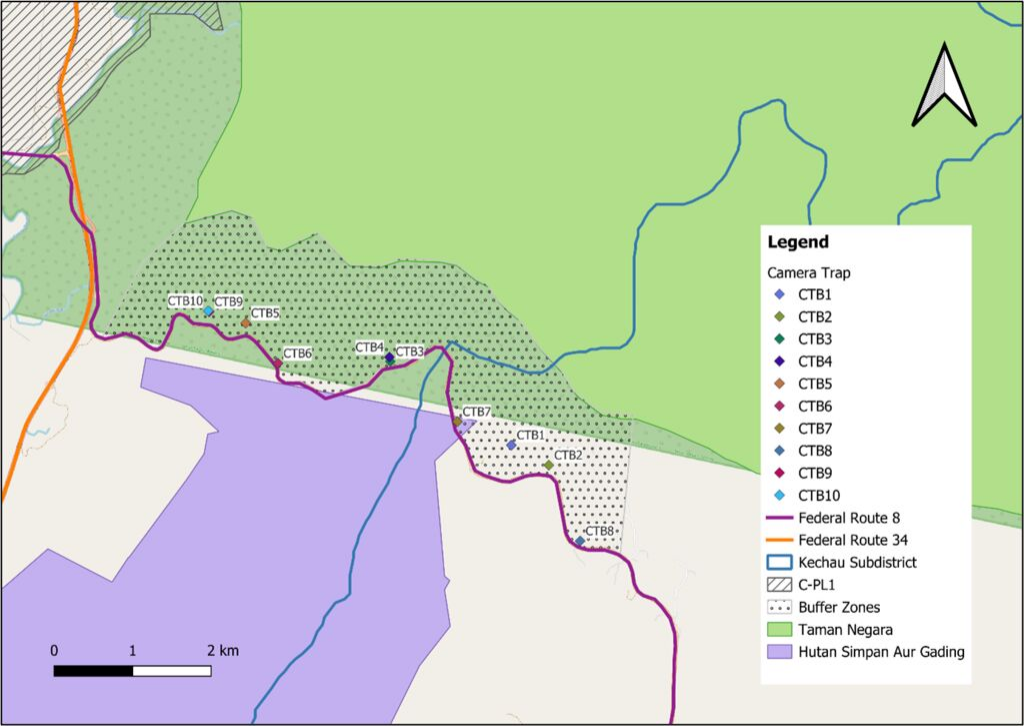
Ten camera traps were deployed at Site B.

Site B (Fig. 4) represents a portion of Taman Negara’s buffer zone, located to the east of the Lipis Geopark. It lies adjacent to Federal Route 8 (the K. Lumpur–K. Bharu Highway) and borders the Hutan Simpan Aur Gading. Along approximately 10 km of this route, 10 camera traps were deployed within the buffer zone, primarily in areas of primary forest, with some sites bordered by plantations and secondary vegetation and situated far from human settlements.

## Materials and Methods

A total of 20 passive infrared camera traps (Reconyx PC800 Hyperfire Professional IR) were deployed across both sites between February and April 2022. For caves in Site A, camera traps were installed facing the entrance or exit of the caves and fixed onto trees or cave walls. In Site B, the camera traps were fixed on trees, facing the existing animal trails and clearings. Each camera was deployed approximately 25-40 cm above ground. They were positioned to face the open ground area with no trees and bushes to reduce the difficulties of capturing and recording the species (Samejima et al. 2012, Mohd-Azlan and Engkamat 2013, Mohd-Azlan et al. 2018, Mohd-Azlan et al. 2019).

The photo-captured species were then identified using identification keys from the books Field Guide to the Reptiles of South-east Asia by Das (2010), Birds of South-east Asia by Robson (2014) and Field Guide to the Mammals of South-East Asia by Francis (2019).

## Data Analysis

Rarefaction analysis was performed on both sites to assess the sampling adequacy of the study, using the Chao et al. (2016) method implemented in iNEXT Online (https://chao.shinyapps.io/iNEXTOnline/). Species diversity at both sites was assessed using the Shannon diversity index (H'), Simpson’s dominance index (D) and Pielou’s evenness index (E), which describe overall diversity, species dominance and the evenness of species distribution. The relative abundance and activity of wildlife species were estimated using the Photographic Capture Rate Index (PCRI), calculated as the number of independent photographic captures per 100 camera trapnights. PCRI values were interpreted as a proxy for relative abundance, recognising that variations may also reflect differences in species behaviour or detectability (Rovero and Marshall 2009).

## Results

Camera trapping of 1,100 camera trap nights (55 days x 20 camera traps) yielded 7,755 photo images, with 3,979 wildlife images representing 288 occasions (52.174%). Occasions of human or anthropogenic activity, unidentifiable and blank photo images comprising 15.580% (86 occasions), 6.522% (36 occasions) and 25.725% (142 occasions) were excluded from the species composition analysis.

Individuals photo-captured in this study resulted in 30 species: seven aves, 22 mammals and one reptile. The most dominant species photo-captured is *Sus scrofa* (n = 96), followed by *Macaca nemestrina* (n = 88) and *Atherurus macrourus* (n = 49). *Centropus* sp., *Argusianus argus*, *Lophura erythrophthalma*, *Presbytis siamensis*, *Echinosorex gymnurus*, Felidae, *Prionailurus bengalensis*, *Hystrix brachyura*, *Callosciurus notatus*, *Lariscus insignis*, Tragulidae, *Paradoxurus musangus* and Scincidae were recorded as singleton captures.

Table 1 shows the diversity and abundance of vertebrate species in both study sites. Site B has more individuals recorded (n = 244) than Site A (n = 147). Six out of 30 species have been recorded at both sites: *Gallus gallus*, *Macaca fascicularis*, *A. macrourus*, Muridae, *S. scrofa* and *Helarctos malayanus*.

To determine the capture rate of this study, the photographic capture rate index (PCRI) was calculated based on the number of independent photo images (occasions) for each species (Table 1). *S. scrofa* (Eurasian wild pig) is the most photographed species with the highest PCRI value of 14.00 (77 occasions), followed by *A. macrourus* (PCRI = 6.727; 37 occasions) and *M. nemestrina* (PCRI = 5.636; 31 occasions). Fifteen species with only one occasion (PCRI = 0.182) are *Centropus* sp., *A. argus*, *L. erythrophthalma*, *A. javanicus*, *P. siamensis*, *E. gymnurus*, *C. temminckii*, Felidae, *P. bengalensis*, *H. brachyura*, *C. notatus*, *L. insignis*, Tragulidae, *P. musangus* and Scincidae.

Looking at the species conservation status globally, *L. erythrophthalma* is a Critically Endangered species. *M. fascicularis*, *M. nemestrina* and *T. indicus* are globally Endangered, while *A. argus*, *A. javanicus*, *N. nebulosa*, *P. pardus* and *H. malayanus* are Vulnerable in IUCN (2026). *P. siamensis* and *C. temminckii* are Near Threatened species globally. At the same time, the following are listed as Least Concern: *C. indica*, *G. gallus*, *M. caeruleus*, *M. muntjak*, *E. gymnurus*, *P. bengalensis*, *H. brachyura*, *A. macrourus*, *C. notatus*, *L. insignis*, *S. scrofa*, *T. glis*, *T. kanchil* and *P. musangus*.

Additionally, seven species are legally protected by CITES from trade and overexploitation — in Appendix I, with the strictest trade policies are *C. temminckii*, *N. nebulosa*, *P. pardus*, *T. indicus* and *H. malayanus*, while *A. argus* and *P. bengalensis* are in Appendix II of CITES (2025).

**Figure 5.**
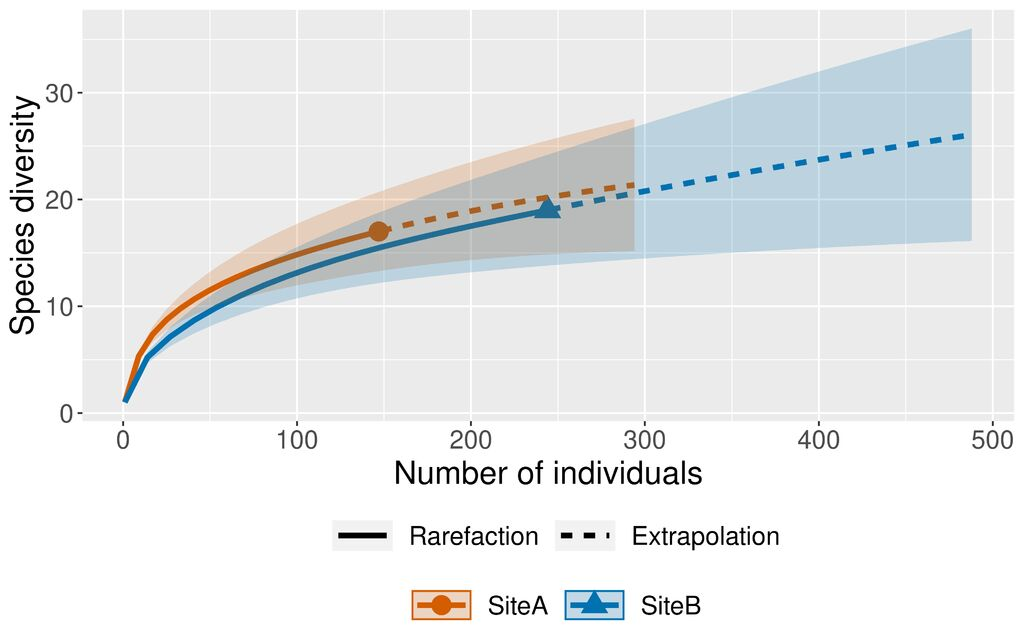
Sample-size-based Rarefaction and Extrapolation Curve for Site A and Site B.

**Figure 6.**
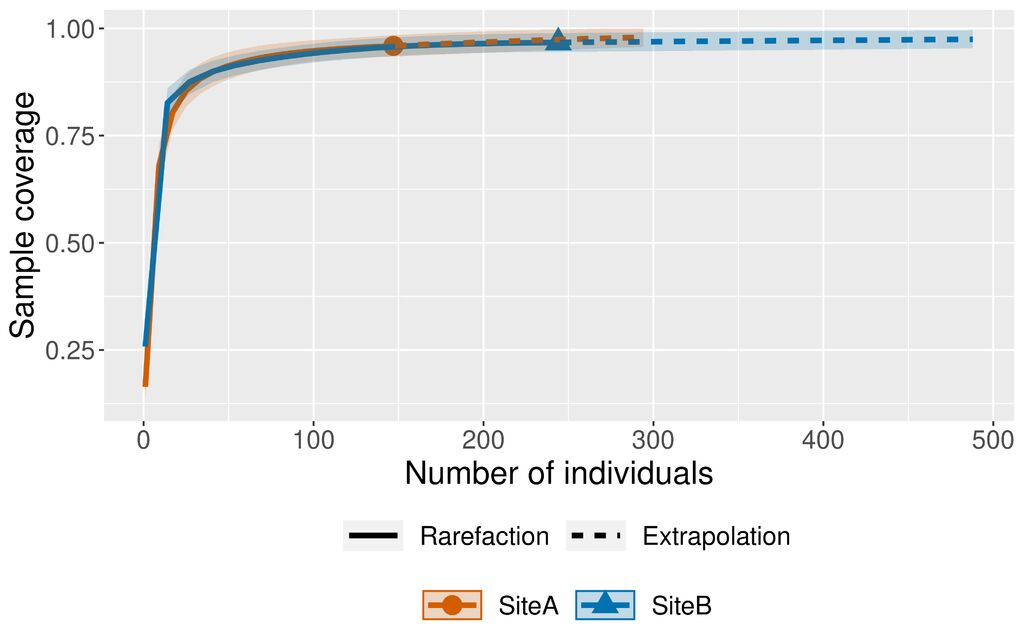
Sample Completeness Curve for Site A and Site B.

**Figure 7.**
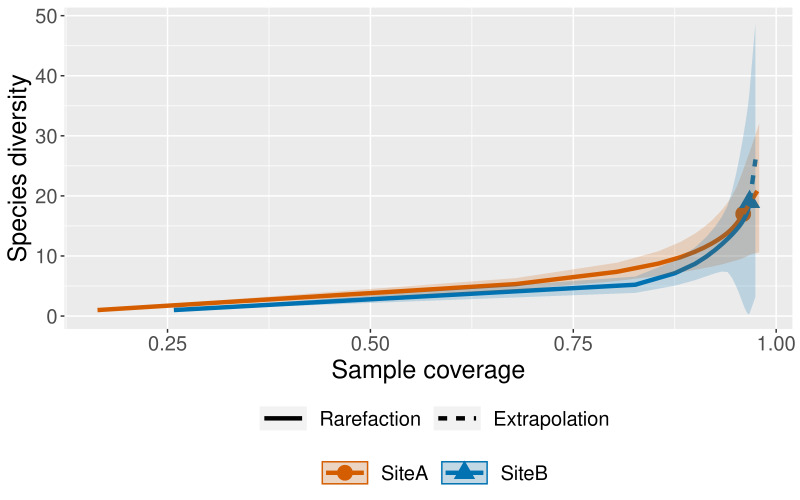
Coverage-based Rarefaction and Extrapolation Curve for Site A and Site B.

Rarefaction and extrapolation analyses revealed distinct patterns between Site A and Site B. The sample-size-based rarefaction and extrapolation curve (Fig. 5) showed that Site A consistently exhibited higher species richness compared to Site B. Despite Site B having a larger number of individuals sampled, its rarefaction curve plateaued at a lower species count, while Site A maintained higher predicted richness. Sample completeness curves (Fig. 6) indicated that both sites achieved high sampling coverage, with Site A and Site B reaching approximately 98% and 99% completeness, respectively. This suggests that the observed differences in species richness are not attributable to undersampling, but rather reflect genuine differences in community composition. Coverage-based rarefaction and extrapolation curves (Fig. 7) showed Site A continued to show higher species richness than Site B. Although confidence intervals overlapped slightly at the highest coverage levels, the overall trend remained consistent, with Site A demonstrating greater diversity.

## Species Account

**Family Felidae**

***Neofelis nebulosa* Griffith, 1821 (Mainland clouded leopard)**

The mainland clouded leopard is one of the wild felids native to Peninsular Malaysia. Based on Chiang and Allen (2017), its population is distributed from the south-eastern Himalayas across Southeast Asia, extending southward into China and Peninsular Malaysia. The mainland clouded leopards are protected in most countries across their range due to population declines dominated by unregulated hunting for pelts, habitat fragmentation and regional declines in habitat quality. In Peninsular Malaysia, the mainland clouded leopard is listed in the Wildlife Conservation Act 2010 (Act 716) (2014) as a Totally Protected Wildlife in the Second Schedule and a Totally Protected Wildlife for the Purpose of Paragraph 68(2)*(c)* in the Tenth Schedule of the Wildlife Conservation (Amendment) Act 2022 (Act A1646) (2022).

A review by Chiang and Allen (2017) has emphasised the versatility of mainland clouded leopards to live in many different habitats, including secondary or selectively logged forests, grasslands and mature evergreen rainforests, with a preference for the dense primary forests. In Peninsular Malaysia, camera-trapping detection has shown a preference of mainland clouded leopards for forested habitats, and increased detection was recorded with increasing altitudes and distance from waterbodies (Chiang and Allen 2017; Kai et al. 2017).

**Figure 8.**
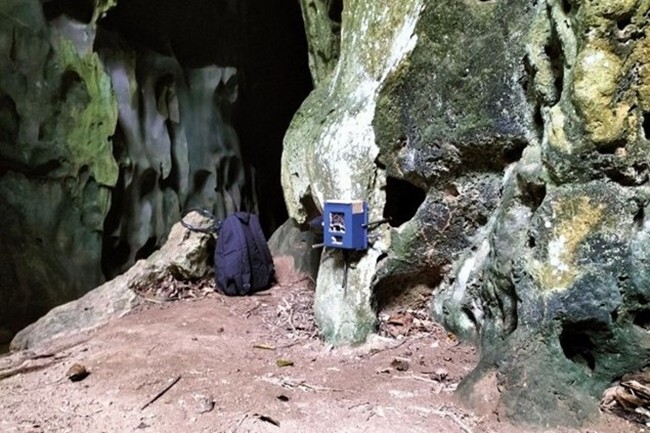
A camera trap was installed at the cave walls facing the cave exit.

**Figure 9.**
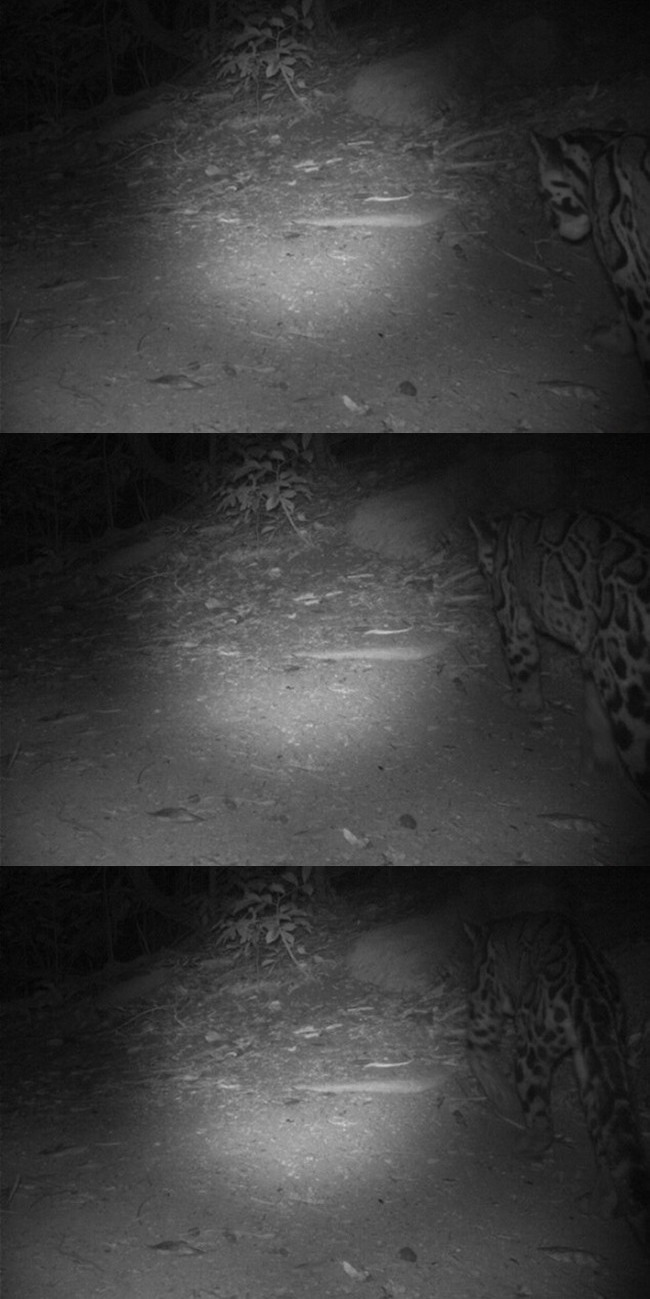
An event showing an individual clouded leopard exiting a cave.

Interestingly, in this study, a camera trap installed facing the trail into a cave (Fig. 8) has captured an individual exiting the cave in Lipis Geopark, as shown in Fig. 9. To date, no study has recorded such activity in Malaysia. However, based on an assessment by Gray et al. (2021), mainland clouded leopard has been recorded occurring in some karst limestone protected areas, for example, the Phou Hin Poun National Protected Area, Laos, to avoid the pressure from illegal hunters. In Vietnam, it is said that this species is likely extinct due to snaring and hunting, and the last documented record was from a protected karst limestone forest in Pu Luong.

## Discussion

There is a notable difference in the number of individuals recorded at both sites, despite the species richness being relatively similar. Site A, which comprises mostly isolated caves, had fewer individuals recorded compared to Site B’s extensive forested areas. Aside from G. Kalong, most caves in Site A showed evidence of human-related disturbances, including tourism, agriculture and guano harvesting. For instance, local guano collection is frequently reported at G. Persit.

Additionally, several caves in Site A offer less favourable conditions for wildlife habitation. Although G. Kalong is devoid of nearby anthropogenic activity, only two species were recorded there. G. Tahi Bintang, despite being located within the C-PL1, yielded no species detections.

Most of the mammal species captured by camera traps in Site A are either typical cave dwellers or generalist species capable of thriving across various environments. Examples include *S. scrofa*, *P. bengalensis* and *P. musangus*, all of which are known for their adaptability and presence even in urban areas (Mohamed et al. 2013, Chua et al. 2015, Francis 2019). Observations of *M. fascicularis* at G. Gunting are consistent with earlier sightings of flocks near G. Gilap in the Gunung Kidul Karst Region (Semiadi et al. 2011). *H. malayanus was* recorded in the Gomantong cave system of the Lower Kinabatangan Wildlife Sanctuary (Jumail and Salgado-Lynn 2021), while *C. notatus* and *T. glis* were noted at Wind Cave, Sarawak and the limestone hills of G. Musang, Kelantan (Thayaparan et al. 2013, Jayaraj et al. 2016). The presence of *E. gymnurus* near G. Keris is likely linked to a river that provides access to amphibian and crustacean prey (Francis 2019, Brunke 2020), while *H. brachyura*, *A. macrourus* and many species of murids are well-known cave inhabitants, utilising these environments primarily for shelter and foraging (Francis 2019, Price 2022).

While avian diversity is severely under-represented in the findings of this study, the species detected provide a glimpse into the avian diversity observed in other research and warrant further investigations. Amongst these, *C. indica* was documented in various karst sites, including Teluk Sumbang in East Kalimantan, Padawan Limestone Area, Sarawak and caves of the Gigantes Islands, Philippines (Bucol et al. 2011, Mansor et al. 2011, Mukhlisi et al. 2021). *A. javanicus*, a highly adaptable species, was recorded in the karst areas of Teluk Sumbang (Roslam et al. 2015, Mukhlisi et al. 2021). *M. caeruleus* was observed in the Lenggong limestone region, known for laying eggs in the nests of other birds and preying on bats (Mansor and Sah 2012, Price 2014, Robson 2014).

Ecosystem dynamics in Lipis Geopark are shaped by predator–prey interactions within its unique karst terrain. Mainland clouded leopards recorded on camera likely act as mesopredators, relying on a diversity of medium‐sized prey (macaques, mousedeers, porcupines and other small carnivores) to persist (Mohamad et al. 2015). These prey species in turn fulfil key ecological functions – for example, arboreal primates disperse seeds and grazing mammals influence understorey vegetation, thereby linking energy flow between canopy and forest floor. The rugged karst environment creates a mosaic of microhabitats (Clements et al. 2006); for instance, macaques and squirrels exploit limestone cliffs and canopy gaps, whereas ground‑dwelling ungulates forage in forest patches around cave entrances. Notably, although clouded leopards are often thought to be strictly arboreal, field evidence from Borneo shows they frequently walk along forest trails and may use caves or rocky ledges as resting sites (Davies and Payne 1982, Rabinowitz et al. 1987, Wilting et al. 2006). Co-existing carnivores (e.g. tigers, leopards, leopard cat, civets) likely avoid direct competition by differing activity times or microhabitat use.

Anthropogenic pressures pose significant threats to Lipis Geopark’s karst biodiversity. Limestone hills in this region are heavily exploited by quarrying and forest conversion (Clements et al. 2006); for example, oil‐palm and rubber plantations have fragmented much of Pahang’s native forest (Clements et al. 2006, Wilting et al. 2006). Even national connectivity plans (such as Malaysia’s Central Forest Spine) leave large gaps: over 4.2 million hectares of forest within the planned linkage network remain unprotected and many corridors have already been lost to development (Wilting et al. 2006). Tourism and extractive use compound these impacts: unmanaged cave visitations can introduce noise, light and litter that disturb bats and other wildlife, while unregulated guano harvesting has been shown to cause bat pup losses and colony abandonment (Debata 2020).

Camera trapping in karst terrain offers both advantages and caveats for wildlife surveys. On one hand, cameras provide continuous, non‐invasive monitoring of cryptic mammals (e.g. large carnivores, ungulates) without requiring human presence. On the other hand, Lipis Geopark’s variable formation and terrain constrain effective camera placement and viewing angle, and units placed at cave mouths may trigger on falling debris or fail to capture animals moving above the sensor line. In our study, we offset these limitations by using multiple trap locations (forest trails, cave entrances). Landscape connectivity is especially important given these detection constraints. Malaysia’s Central Forest Spine Master Plan explicitly aims to link forests (Wilting et al. 2006), but many proposed corridors in Pahang remain unprotected or degraded. In the Lipis Geopark’s landscape, conserving or restoring forested linkages – for example, riparian corridors along rivers or continuous canopy on ridgelines – would facilitate movements of clouded leopards and other wide‐ranging species and integrate the Geopark with surrounding reserves. The conservation value of corridors is well‐documented: a meta-analysis found that connected habitat patches increase movement of non‐avian vertebrates by roughly 50% compared to isolated fragments (Gilbert-Norton et al. 2010). By integrating these wildlife corridors into geopark planning (complementing core protections), Lipis Geopark can function as part of a broader connected landscape, maintaining gene flow and long‐term population viability of its species (Wilting et al. 2006, Gilbert-Norton et al. 2010).

## Conclusion

This study offers critical baseline data on the terrestrial vertebrate diversity in Lipis Geopark, Pahang, with a specific focus on the cave ecosystems of Merapoh and the adjacent forested buffer zones of Taman Negara. Across both sites, camera traps documented 30 species, including 11 species categorised as threatened under the IUCN (2026) and seven listed under CITES (2025). The cave complex (Site A) exhibited higher species diversity and evenness, despite fewer individual detections than in the buffer zones (Site B). This suggests that karst caves may serve as biodiversity reservoirs, particularly for species sensitive to human disturbance or seeking specialised habitats.

Of particular ecological significance is the novel documentation of a mainland clouded leopard (*Neofelis nebulosa*) utilising a cave — a rare behaviour that expands our understanding of this elusive carnivore’s ecological plasticity. This behaviour may reflect adaptive responses to anthropogenic pressures or novel foraging and shelter strategies. Given the vulnerability of karst habitats to quarrying, agriculture and unregulated tourism, this observation strongly supports the need for urgent protective measures targeting key cave sites and their surrounding forest matrices.

Future studies should prioritise comprehensive cave biodiversity assessments across the unexplored limestone systems in Merapoh and other sectors of Lipis Geopark. Similarly, expanded camera trapping in additional buffer zones will refine our understanding of their conservation value and species composition. Integrating these findings into landscape-level conservation frameworks — such as the Central Forest Spine ecological network — will be essential to maintain habitat connectivity, safeguard rare species and ensure the long-term ecological integrity of the Lipis Geopark.

## Figures and Tables

**Table 1.** Species recorded throughout the study.

**No**.	**Species**	**Common name**	**IUCN (2026)**	**CITES (2025)**	**Number of individuals**		**Occasions**	**PCRI**
					**Site A**	**Site B**		
** Aves **								
** Cuculidae **								
1	*Centropus* sp.	Coucal	-	-	0	1	1	0.182
** Columbidae **								
2	*Chalcophaps indica*	Asian emerald dove	LC	-	5	0	4	0.727
** Phasianidae **								
3	*Argusianus argus*	Great argus	VU	II	0	1	1	0.182
4	*Gallus gallus*	Red junglefowl	LC	-	2	1	3	0.545
5	*Lophura erythrophthalma*	Crestless fireback	CR	-	0	1	1	0.182
** Muscicapidae **								
6	*Myophonus caeruleus*	Blue whistling-thrush	LC	-	22	0	20	3.636
** Sturnidae **								
7	*Acridotheres javanicus*	Javan myna	VU	-	2	0	1	0.182
**Mammals**								
** Cercopithecidae **								
8	*Macaca fascicularis*	Long-tailed macaque	EN	-	5	5	6	1.091
9	*Macaca nemestrina*	Southern pig-tailed macaque	EN	-	0	88	31	5.636
10	*Presbytis siamensis*	Pale-thighed langur	NT	-	0	1	1	0.182
** Cervidae **								
11	*Muntiacus muntjak*	Red muntjac	LC	-	0	23	21	3.818
** Erinaceidae **								
12	*Echinosorex gymnurus*	Moonrat	LC	-	1	0	1	0.182
** Felidae **								
13	*Catopuma temminckii*	Asian golden cat	VU	I	0	2	1	0.182
14	Felidae	Unidentified felid	-	-	0	1	1	0.182
15	*Neofelis nebulosa*	Mainland clouded leopard	VU	I	3	0	3	0.545
16	*Panthera pardus*	Leopard	VU	I	0	4	4	0.727
17	*Prionailurus bengalensis*	Leopard cat	LC	II	1	0	1	0.182
** Hystricidae **								
18	*Hystrix brachyura*	Malayan porcupine	LC	-	1	0	1	0.182
19	*Atherurus macrourus*	Asiatic brush-tailed porcupine	LC	-	46	3	37	6.727
** Muridae **								
20	Muridae	Unidentified rats and mice	-	-	13	5	17	3.091
** Sciuridae **								
21	*Callosciurus notatus*	Plantain squirrel	LC	-	1	0	1	0.182
22	*Lariscus insignis*	Three-striped ground squirrel	LC	-	0	1	1	0.182
** Suidae **								
23	*Sus scrofa*	Eurasian wild pig	LC	-	13	83	77	14.000
** Tapiridae **								
24	*Tapirus indicus*	Asian tapir	EN	I	0	4	4	0.727
** Tupaiidae **								
25	*Tupaia glis*	Common treeshrew	LC	-	25	0	23	4.182
** Tragulidae **								
26	Tragulidae	Unidentified mousedeer	-	-	0	1	1	0.182
27	*Tragulus kanchil*	Lesser mousedeer	LC	-	0	3	3	0.545
** Ursidae **								
28	*Helarctos malayanus*	Sun bear	VU	I	5	16	20	3.636
** Viverridae **								
29	*Paradoxurus musangus*	Common palm civet	LC	-	1	0	1	0.182
**Reptiles**								
** Scincidae **								
30	Scincidae	Unidentified skink	-	-	1	0	1	0.182
	Total individuals (N)				147	244		
	Total number of species				17	19		
	Dominance (D')				0.169	0.261		
	Evenness (J')				0.749	0.597		
	Shannon-Wiener index (H')				2.123	1.758		

**Keys**; **Site A**: Merapoh cave complex; **Site B**: Taman Negara’s buffer zones; **IUCN (2026)**: IUCN Red List of Threatened Species status, **CR**: Critically Endangered, **EN**: Endangered, **VU**: Vulnerable, **NT**: Near Threatened and **LC**: Least Concern and **-**: Absent; **CITES (2025)**, listed in Convention on International Trade in Endangered Species of Wild Flora and Vertebrates, **I**: Appendix I, **II**: Appendix II and **-**: Absent; **Occasions**; number of independent photo images; **PCRI**: photographic capture rate index of each species (ratio of independent photo images captured by the camera traps to the number of trap days (number of complete 24-hour periods during which cameras operate until the film was full or camera was retrieved) which then will be multiplied by 100).
